# Improving access to interventions among mothers screened positive for post-partum depression (PPD) at National Programme on Immunization (NPI) clinics in south-western and south-eastern Nigeria – A service development report

**DOI:** 10.19185/matters.201707000005

**Published:** 2017-10-12

**Authors:** Muideen O Bakare, Mashudat A Bello-Mojeed, Kerim M Munir, Olaniyi O Duduyemi, Andrew O Orovwigho, Odutola I Odetunde, Olufemi G Taiwo, Jushua A Olofinlade, Olakunle N Omotoso, Olayinka H Famurewa, Oladipupo O Omolabi, Adebayo O Jejeloye

**Affiliations:** Childhood Neuropsychiatric Disorders Initiatives, Child and Adolescent Unit, Federal Neuropsychiatric Hospital, Enugu; Childhood Neuropsychiatric Disorders Initiatives, Child and Adolescent Centre, Federal Neuropsychiatric Hospital, Lagos; University Center for Excellence in Developmental Disabilities, Boston Children Hospital; Childhood Neuropsychiatric Disorders Initiatives, Federal Neuropsychiatric Hospital, Enugu, Child and Adolescent Unit, Federal Neuropsychiatric Hospital, Enugu; Child and Adolescent Unit, Federal Neuropsychiatric Hospital, Enugu; Department of Paediatric, University of Nigeria Teaching Hospital; Childhood Neuropsychiatric Disorders Initiatives, Childhood Neuropsychiatric Disorders Initiatives, Child and Adolescent Unit, Federal Neuropsychiatric Hospital; Childhood Neuropsychiatric Disorders Initiatives, Childhood Neuropsychiatric Disorders Initiatives, Child and Adolescent Centre, Federal Neuropsychiatric Hospital, Lagos; Childhood Neuropsychiatric Disorders Initiatives, Enugu State University of Science and Technology, Child and Adolescent Centre, Federal Neuropsychiatric Hospital

**Keywords:** Intervention, Human Centred Design, Post-Partum Depression, Immunization, Infant Growth

## Abstract

We investigate the possibility of improving access to interventions among mothers screened positive for post-partum depression (PPD) at National Programme on Immunization (NPI) clinics randomly selected from Lagos and Enugu States in south-western and south-eastern Nigeria respectively. The principle of human centred design was employed by engaging the mothers screened positive for PPD to be part of the decision making regarding their further assessment and intervention services. The study brought intervention services to primary healthcare centre at the NPI clinics. Improvement in willingness to seek interventions was observed among the mothers screened positive for PPD in this study when compared to our observation in a previous report, where mothers diagnosed with PPD were referred and requested to visit a mental health facility closer to their NPI clinics for further assessment and interventions (95.2% versus 33.7%). Interventional services for the mothers diagnosed with PPD also impact positively on the growth parameters of their infants on follow-up. Principle of human centred design improved access to intervention services among the mothers and infants studied. NPI clinics at primary healthcare level would provide appropriate forum for early screening of mothers for PPD and interventions in low-resource setting like Nigeria. There would be improvement in maternal and child health coverage if the Nigerian Government can adapt human centred design principles employed in this study nationwide.

## Introduction

An earlier report described the attempt made at introducing depression and developmental screenings into the primary healthcare level of the National Programme on Immunization (NPI) in South-Eastern Nigeria [1]. This report also documented association between maternal postpartum depression and growth parameters of weight and length among the infants of mothers studied. Mothers diagnosed with postpartum depression during the study were referred to Federal Neuropsychiatric Hospital, Enugu, Nigeria (a Mental Health Facility) for further assessment and interventional follow-up. However, out of a total of 101 mothers diagnosed with depression in the study and referred for further assessment and interventional follow-up, only 34 (33.7%) eventually reported for further evaluation and interventional follow-up. The rest were lost to follow-up after the screening process [1].

In response to the poor access to interventions by these mothers with post-partum depression as reflected by the above finding, probably due to stigma associated with visiting mental health facility in this environment, a study was set up using the principle of human centred design.

The study that was conducted in two phases of **Piloting** and **Prototyping** has the overall objective of improving access of mothers screened positive for postpartum depression to interventions and by that resolve their symptoms of depression, improve growth outcome in their infants and promote mother-child bonding.

## Objective

The objective of the study was to improve access of mothers screened positive for post-partum depression to interventions and by that resolve their symptoms of depression, improve growth outcome in their infants and promote mother-child bonding.

## Results & Discussion

### First phase (Piloting phase)

The piloting phase engaged the women and made them to actively participate in decision making regarding their further assessment and subsequent follow-up treatment (Appendix 1) and asked the following questions:

- How do we get more mothers to consent to intervention services?
- How do we communicate screening outcome to mothers in a way not to confer a feeling of being stigmatized?
- Where would the women screened positive for PPD prefer for further assessment and possible follow-up for interventions?

Two hundred and seventeen (217) women were screened for post-partum depression in the piloting phase of the study.

- Fifty seven (26.3%) of the 217 mothers screened had a cut-off point of 7 and above on EPDS and therefore screened positive for PPD and were administered the Appointment Booking Form (Appendix 1).
- Majority of the women screened positive for PPD, 47 (82.5%) of 57 opted for further assessment and possible follow-up treatment at NPI clinics as against the option of Home visit intervention.
- Many of the women, 54 (94.7%) of 57 opted for option of group therapy if they are going to be involved in further interventional follow-up.

### Second phase (Prototyping phase)

The outcome in piloting phase of the study provided the template for full launch into second phase of the study, where further assessment and interventional follow-up focused largely on NPI clinics, following the baseline screening with EPDS. The location of the study was further extended to Enugu State in South-Eastern Nigeria during this phase.

In addition to the women screened at piloting phase, a total of 3,686 women were screened at the selected NPI clinics, 958 (26%) had a score of 7 and above on EPDS and were involved in further follow-up assessment. On further follow-up assessment using both clinical judgment and depressive module of MINI, 674 women (18%) met diagnostic criteria for post-partum depression.

The age range of the mothers with diagnosis of postpartum depression was between 22 and 35 years, with a mean age of 29.0 ± 3.8 years. The age range of their infants was between 0.5 and 3.5 months, with a mean age of 2.55 ± 0.91 months. The sex distribution of the infants were 405 females (60.1%) and 269 males (39.9%). The mean EPDS score at baseline was 11.2 ± 5.5.

### Interpretation of Results

Maternal Postpartum depression (PPD) was diagnosed in the mothers screened, when their infants were average age of about three months (3 Months) {Table 1}. Growth parameters in the infants deteriorated over the following three months at initiation of interventions for the mothers’ depressive symptoms, when the infants were at about average age of six months (6 Months) {Table 2}. Following further interventions, the growth parameters of the infants had peaked over the subsequent six months, when the average age of the children was about nine months (9 Months) and the peak had been sustained {Table 3}. The fourth follow-up impact assessment, nine months after the baseline showed closer correlations with the standard WHO growth charts, the average age of the children at this stage was about twelve months (12 Months) {Table 4}. With each follow-up impact assessment, few of the mothers were being lost to follow up, which may necessitate the need for remote interface for the purpose of assessment and interventions in future study design.

From the foregoing, a total of 95.2% of the mothers diagnosed with post-partum depression in this study accessed interventions until the fourth and last impact assessment follow-up period, when the average age of their infants was about 12 months. This result is an improvement over the observation made in our previous report, where only 33.7% of the women diagnosed with post-partum depression showed up for further assessment and interventions following referral to a mental health facility [1].

### Discussion

#### Main Findings

The present study showed an improvement in the percentage of women willing to seek intervention, if the intervention is taking to primary care level at immunization clinics, as against referral to mental healthcare facility, where the mothers may have perceived stigma visiting.

More women were likely to consent to treatment and intervention if they are engaged and made to be part of the decision making in their own treatment. Follow-up assessment and interventions for the mothers showed a positive impact on the growth parameters of the infants over time.

#### Immunization clinics and intervention for maternal PPD

Immunization clinics at the primary healthcare centers had been earlier noted to constitute major clinical source of convergence for mothers and their infants and might provide a good forum for screening for depression in the mothers and developmental concerns in the children [1] [2] and may also provide a right forum for providing interventions [1] [2]. It is along this assumption that the idea behind the present study was tested.

#### Human centred design and interventional follow-up

Human centred design is defined as a creative approach to interactive systems development that aims to make systems usable and useful by focusing on the users, designing around their needs and requirements at all stages, and by applying human factors/ergonomics, usability knowledge, and techniques. This approach enhances effectiveness and efficiency, improves human well-being, user satisfaction, accessibility and sustainability; and counteracts possible adverse effects of use on human health, safety and performance [3] [4].

The principle of human centred design was employed in the piloting and prototyping phase of this study by engaging the women and making them to actively participate in determining the course of their own interventions and treatment. This principle probably went a long way in promoting the willingness of the women to seek interventions, which also in turn served the ultimate benefit of promoting the growth and development of their infants as reflected by the findings of this study.

#### Maternal PPD and infant growth

Previous studies [1] [5] [6] [7] [8] [9] [10] [11] [12] [13] had documented association between maternal depressive symptoms and child stunting. Observation in this study showed that the interventional follow-up of the mothers produced improvement in growth parameters of their infants followed up in this study.

## Conclusions

Addressing maternal PPD through early detection and interventions would improve optimal development of infants in low resource countries like Nigeria. Principle of human centred design improved access to intervention services among the mothers and infants studied. NPI clinics would provide appropriate forum for early screening of mothers for PPD and interventions in low-resource setting like Nigeria. We are of the opinion that there would be improvement in maternal and child health care coverage if the Nigerian Government can adapt human centred design principles employed in this study nationwide.

## Supplementary Material

Appendix 1 Appendix 1: Submitted as supplementary information file.

## Figures and Tables

**Table 1:** Baseline at the point of First follow-up Assessment Showed the growth parameters of the infants of the mothers at first follow-up assessment (baseline) compared to WHO Standard Growth Charts at 50th percentile.

Parameters	Total Number of Cases N (%)	Mothers EPDS Mean Score (N/30)	Child Weight (Kg)	Child Length (Cm)	Child Head Circumference (Cm)
	674 (100)	11.2 ± 5.5			
Measured (Mean)			5.02 ± 0.99	54.30 ± 4.40	38.85 ± 2.52
Mean at Standardized 50^th^ Percentile			5.83 ± 0.86	59.40 ± 2.95	39.25 ± 1.85
Correlation (r)			r = 0.77	r = 0.79	r = 0.84
Goodness of Fit (R^2^)			R^2^ = 0.59	R^2^ = 0.62	R^2^ = 0.70

**Table 2:** Second Follow-up Assessment (approximately three months after the baseline) Showed the growth parameters of the infants at second follow-up impact assessment, approximately three months after the baseline. Twenty (3.0%) of the mothers and their infants had been lost to follow-up at the NPI Clinics.

Parameters	Total Number of Cases N (%)	Mothers EPDS Mean Score (N/30)	Child Weight (Kg)	Child Length (Cm)	Child Head Circumference (Cm)
	654 (100)	5.9 ± 4.5			
Measured (Mean)			6.95 ± 0.51	62.40 ± 2.80	41.30 ± 1.95
Mean at Standardized 50^th^ Percentile			7.39 ± 0.50	66.20 ± 1.75	42.25 ± 0.75
Correlation (r)			r = 0.61	r = 0.28	r = 0.25
Goodness of Fit (R^2^)			R^2^ = 0.38	R^2^ = 0.08	R^2^ = 0.06

**Table 3:** Third Follow-up Assessment (approximately six months after the baseline) Showed the growth parameters of the infants at third follow-up impact assessment, approximately six months after the baseline. Thirty two (4.8%) of the mothers and their infants had been lost to follow-up at the NPI Clinics.

Parameters	Total Number of Cases N (%)	Mothers EPDS Mean Score (N/30)	Child Weight (Kg)	Child Length (Cm)	Child Head Circumference (Cm)
	642 (100)	3.9 ± 1.4			
Measured (Mean)			8.04 ± 0.55	67.90 ± 3.14	43.75 ± 0.92
Mean at Standardized 50^th^ Percentile			8.41 ± 3.8	70.50 ± 1.43	44.30 ± 0.67
Correlation (r)			r = 0.78	r = 0.80	r = 0.85
Goodness of Fit (R^2^)			R^2^ = 0.0.61	R^2^ = 0.64	R^2^ = 0.72

**Table 4:** Fourth and Last Follow-up Assessment (approximately nine months after the baseline) Showed the growth parameters of the infants at fourth and last follow-up impact assessment, approximately nine months after the baseline. Thirty two (4.8%) of the mothers and their infants had been lost to follow-up at the NPI Clinics.

Parameters	Total Number of Cases N (%)	Mothers EPDS Mean Score (N/30)	Child Weight (Kg)	Child Length (Cm)	Child Head Circumference (Cm)
	642 (100)	1.9 ± 0.6			
Measured			8.92 ± 0.55	74.10 ± 1.60	45.50 ± 0.53
Mean at Standardized 50^th^ Percentile			9.30 ± 0.41	74.20 ± 1.40	45.45 ± 0.50
Correlation (r)			r = 0.89	r = 0.99	r = 0.74
Goodness of Fit (R^2^)			R^2^ = 0.80	R^2^ = 0.97	R^2^ = 0.55
