# Supplementary material for: Improving access to interventions among mothers screened positive for post-partum depression (PPD) at National Programme on Immunization (NPI) clinics in south-western and south-eastern Nigeria – A service development report
Source: Matters (Zur). Author manuscript; Available in PMC 2017 Nov 12. (PMC5665652; doi:10.19185/matters.201707000005)
Supplement: Appendix 1 — Submitted as supplementary information file. [file NIHMS913834-supplement-Appendix_1.pdf]

Appendix 1: Appointment Booking Form

WE LOOK FORWARD TO SEEING YOU

Dear Mother,

During today's screening, we found that you have some features of depression.

We would like to offer you some help to resolve this, so you can provide the best possible care for your child, and be healthy yourself.

If you are interested in getting help, you can use this form to choose a time and place for your first appointment.

We look forward to supporting you and your child. If you have any questions, call **08028140458**.

Thank you!

1 SELECT APPOINTMENT LOCATION & TIME

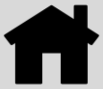

☐ Home Support

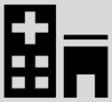

☐ Individual Support at the NPI

DAY ____________ **TIME** ____________

2 SUPPORT GROUP

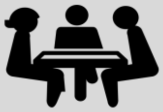

Group Support at the NPI

☐ YES ☐ NO

---

3 YOUR INFO

NAME / ID ____________

PHONE ____________

ADDRESS ____________

____

4 FOR OFFICE RECORD

LOCATION

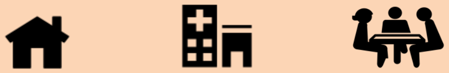

☐ ☐ ☐

DAY ____________

TIME ____________
